# Bis(acetonitrile-κ*N*)dichlorido(η^4^-cyclo­octa-1,5-diene)ruthenium(II) acetonitrile monosolvate

**DOI:** 10.1107/S1600536811027723

**Published:** 2011-07-16

**Authors:** Haleden Chiririwa, Reinout Meijboom, Samson O. Owalude, Uche B. Eke, Charmaine Arderne

**Affiliations:** a Department of Chemistry, University of Johannesburg, PO Box 524, Auckland Park, Johannesburg 2006, South Africa; b Department of Chemistry, University of Ilorin, P M B 1515, Ilorin, Nigeria

## Abstract

In the title Ru^II^ complex, [RuCl_2_(C_8_H_12_)(C_2_H_3_N)_2_]·CH_3_CN, the metal ion is coordinated to the centers of each of the double bonds of the cyclo­octa­diene ligand, to two chloride ions (in *cis* positions) and to two N-atom donors (from MeCN mol­ecules) that complete the coordination sphere for the neutral complex. The coordination about the Ru^II^ atom can thus be considered to be octa­hedral with a slightly trigonal distortion. There is also one acetonitrile solvent mol­ecule per mol­ecule which is outside the coordination sphere of the ruthenium atom.

## Related literature

For the structure of the water solvate complex, see: Ashworth *et al.* (1987 ▶).
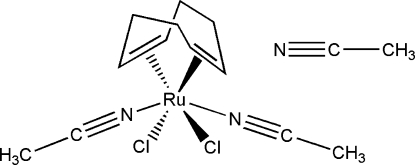


## Experimental

### 

#### Crystal data

[RuCl_2_(C_8_H_12_)(C_2_H_3_N)_2_]·C_2_H_3_N
*M* *_r_* = 403.31
Monoclinic, 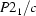

*a* = 8.7033 (3) Å
*b* = 7.2434 (3) Å
*c* = 26.4178 (10) Å
β = 95.903 (1)°
*V* = 1656.59 (11) Å^3^
*Z* = 4
Mo *K*α radiation
μ = 1.26 mm^−1^
*T* = 100 K
0.32 × 0.20 × 0.13 mm

#### Data collection

Bruker APEXII CCD diffractometer
Absorption correction: numerical (AXScale; Bruker, 2010 ▶) *T*_min_ = 0.688, *T*_max_ = 0.853
16175 measured reflections
4125 independent reflections
3806 reflections with *I* > 2σ(*I*)
*R*_int_ = 0.030

#### Refinement

*R*[*F* ^2^ > 2σ(*F* ^2^)] = 0.022
*wR*(*F* ^2^) = 0.054
*S* = 1.07
4125 reflections
184 parameters
H-atom parameters constrained
Δρ_max_ = 0.67 e Å^−3^
Δρ_min_ = −0.61 e Å^−3^

### 

Data collection: *APEX2* (Bruker, 2010 ▶); cell refinement: *SAINT* (Bruker, 2010 ▶); data reduction: *SAINT*; program(s) used to solve structure: *SHELXS97* (Sheldrick, 2008 ▶); program(s) used to refine structure: *SHELXL97* (Sheldrick, 2008 ▶); molecular graphics: *OLEX2* (Dolomanov *et al.*, 2009 ▶); software used to prepare material for publication: *publCIF* (Westrip, 2010 ▶).

## Supplementary Material

Crystal structure: contains datablock(s) I, global. DOI: 10.1107/S1600536811027723/go2018sup1.cif

Structure factors: contains datablock(s) I. DOI: 10.1107/S1600536811027723/go2018Isup2.hkl

Additional supplementary materials:  crystallographic information; 3D view; checkCIF report

## Figures and Tables

**Table 1:** Selected bond lengths (Å)

Ru1—N1	2.0303 (15)
Ru1—N2	2.0418 (15)
Ru1—C1	2.2082 (16)
Ru1—C8	2.2116 (17)
Ru1—C4	2.2154 (17)
Ru1—C5	2.2225 (17)
Ru1—Cl1	2.4212 (4)
Ru1—Cl2	2.4265 (4)

**Fig. 1.**
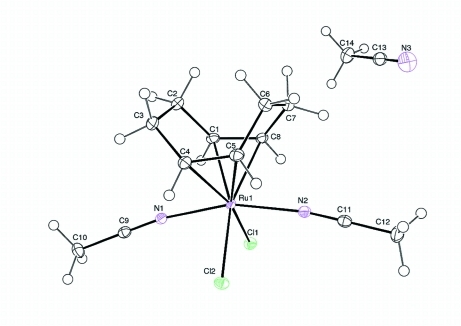
A perspective view of C12H18Cl2N2Ru.C2H3N (1) showing the atomic numbering scheme.

## supplementary crystallographic information

### Comment

The present ruthenium complex, Fig.1, has been synthesized earlier (Ashworth *et al.* 1987). The structure obtained by Ashworth *et al.* was a room temperature determination and with a water molecule as solvate. The current low temperature determination presents an acetonitrile molecule in the crystal lattice that is outside the coordination sphere of the ruthenium. To the best of our knowledge, there are no reports of other structures determined with organonitriles, obtained from the ruthenium [{RuCl_2_(COD)}*_x_*] polymer. Organonitriles have been used over many years in synthethic inorganic chemistry and an interest in the chemistry of metal-nitrile complexes has prompted several reviews.

The two acetonitrile ligands are not *trans* to each other, as the N(1)—Ru—N(2) angle is 163.15 (6)°. This is due to repulsion by the alkene bonds of the COD ligand. It would seem that one of the acetonitrile ligands is slightly bent. The N(1)—C(9)—C(10) bond angle is 179.11 (19)°, whereas the same angle for the other acetonitrile is 178.3 (2)°. This is due to packing forces.

### Experimental

A suspension of [{RuCl_2_(COD)}*_x_*] (0.5 g) in acetonitrile (20 ml) was refluxed for 6 h. The orange solution was filtered hot and concentrated on a steam bath to half volume and cooled to 0° C overnight affording orange crystals suitable for X-ray diffraction studies.

### Refinement

Hydrogen atoms could be identified from the difference Fourier map but once these atoms were refined, their distances from the parent atoms were found to be significantly shorter than the ideal distances for C—H and N—H respectively. The H-atoms were therefore geometrically positioned and refined in the riding-model approximation, with C—H = 0.97 Å, N—H = 0.89 Å, and *U*_iso_(H) = 1.2Ueq(C) or 1.5Ueq(N). For (I), the highest peak in the final difference map is 0.55Å from H1 and the deepest hole is 0.22Å from H5.

### Crystal data

**Table:**

[RuCl_2_(C_8_H_12_)(C_2_H_3_N)_2_]·C_2_H_3_N	*F*(000) = 816
*M**_r_* = 403.31	*D*_x_ = 1.617 Mg m^−^^3^
Monoclinic, *P*2_1_/*c*	Mo *K*α radiation, λ = 0.71073 Å
Hall symbol: -P 2ybc	Cell parameters from 9251 reflections
*a* = 8.7033 (3) Å	θ = 2.7–28.3°
*b* = 7.2434 (3) Å	µ = 1.26 mm^−^^1^
*c* = 26.4178 (10) Å	*T* = 100 K
β = 95.903 (1)°	Block, orange
*V* = 1656.59 (11) Å^3^	0.32 × 0.20 × 0.13 mm
*Z* = 4	

### Data collection

**Table:**

Bruker APEXII CCD diffractometer	4125 independent reflections
Radiation source: fine-focus sealed tube	3806 reflections with *I* > 2σ(*I*)
graphite	*R*_int_ = 0.030
φ and ω scans	θ_max_ = 28.3°, θ_min_ = 1.6°
Absorption correction: numerical (AXScale; Bruker, 2010)	*h* = −11→11
*T*_min_ = 0.688, *T*_max_ = 0.853	*k* = −6→9
16175 measured reflections	*l* = −35→35

### Refinement

**Table:**

Refinement on *F*^2^	Primary atom site location: structure-invariant direct methods
Least-squares matrix: full	Secondary atom site location: difference Fourier map
*R*[*F*^2^ > 2σ(*F*^2^)] = 0.022	Hydrogen site location: inferred from neighbouring sites
*wR*(*F*^2^) = 0.054	H-atom parameters constrained
*S* = 1.07	*w* = 1/[σ^2^(*F*_o_^2^) + (0.0202*P*)^2^ + 1.4236*P*] where *P* = (*F*_o_^2^ + 2*F*_c_^2^)/3
4125 reflections	(Δ/σ)_max_ = 0.001
184 parameters	Δρ_max_ = 0.67 e Å^−^^3^
0 restraints	Δρ_min_ = −0.61 e Å^−^^3^

### Special details

**Table:**

Geometry. All e.s.d.'s (except the e.s.d. in the dihedral angle between two l.s. planes) are estimated using the full covariance matrix. The cell e.s.d.'s are taken into account individually in the estimation of e.s.d.'s in distances, angles and torsion angles; correlations between e.s.d.'s in cell parameters are only used when they are defined by crystal symmetry. An approximate (isotropic) treatment of cell e.s.d.'s is used for estimating e.s.d.'s involving l.s. planes.
Refinement. Refinement of *F*^2^ against ALL reflections. The weighted *R*-factor *wR* and goodness of fit *S* are based on *F*^2^, conventional *R*-factors *R* are based on *F*, with *F* set to zero for negative *F*^2^. The threshold expression of *F*^2^ > σ(*F*^2^) is used only for calculating *R*-factors(gt) *etc*. and is not relevant to the choice of reflections for refinement. *R*-factors based on *F*^2^ are statistically about twice as large as those based on *F*, and *R*- factors based on ALL data will be even larger. The Following Model and Quality ALERTS were generated - (Acta-Mode) <<< Format: alert-number_ALERT_alert-type_alert-level text 912_ALERT_4_C Missing # of FCF Reflections Above STh/*L*= 0.600 5 Noted.

### Fractional atomic coordinates and isotropic or equivalent isotropic displacement parameters (Å^2^)

**Table:**

	*x*	*y*	*z*	*U*_iso_*/*U*_eq_	
Ru1	0.732203 (14)	0.145839 (19)	0.109901 (5)	0.01058 (5)	
Cl1	0.77031 (5)	0.38454 (6)	0.048264 (16)	0.01597 (9)	
Cl2	1.00549 (5)	0.09785 (6)	0.134734 (15)	0.01607 (9)	
N1	0.76802 (16)	−0.0274 (2)	0.05184 (5)	0.0132 (3)	
N2	0.75889 (16)	0.3467 (2)	0.16432 (6)	0.0145 (3)	
N3	0.2246 (2)	0.6581 (3)	0.20027 (7)	0.0349 (5)	
C1	0.49326 (19)	0.1021 (3)	0.07512 (7)	0.0152 (3)	
H1	0.5348	0.1237	0.0438	0.018*	
C2	0.4309 (2)	−0.0888 (3)	0.08525 (7)	0.0180 (4)	
H2A	0.3862	−0.1427	0.0526	0.022*	
H2B	0.3464	−0.0762	0.1074	0.022*	
C3	0.5526 (2)	−0.2234 (3)	0.11060 (7)	0.0180 (4)	
H3A	0.5015	−0.3090	0.1328	0.022*	
H3B	0.5947	−0.2980	0.0838	0.022*	
C4	0.6854 (2)	−0.1285 (2)	0.14209 (7)	0.0157 (3)	
H4	0.7877	−0.1611	0.1361	0.019*	
C5	0.6665 (2)	0.0024 (3)	0.17886 (6)	0.0159 (3)	
H5	0.7561	0.0577	0.1961	0.019*	
C6	0.5090 (2)	0.0627 (3)	0.19318 (7)	0.0183 (4)	
H6A	0.5192	0.0962	0.2297	0.022*	
H6B	0.4372	−0.0433	0.1885	0.022*	
C7	0.4375 (2)	0.2278 (3)	0.16207 (7)	0.0181 (4)	
H7A	0.3237	0.2144	0.1583	0.022*	
H7B	0.4630	0.3429	0.1813	0.022*	
C8	0.49209 (19)	0.2461 (3)	0.10961 (7)	0.0153 (3)	
H8	0.5277	0.3635	0.0998	0.018*	
C9	0.8070 (2)	−0.1171 (3)	0.02039 (6)	0.0145 (3)	
C10	0.8594 (2)	−0.2322 (3)	−0.01961 (7)	0.0189 (4)	
H10A	0.9666	−0.2698	−0.0100	0.028*	
H10B	0.8531	−0.1621	−0.0515	0.028*	
H10C	0.7938	−0.3421	−0.0242	0.028*	
C11	0.79305 (19)	0.4484 (3)	0.19626 (6)	0.0150 (3)	
C12	0.8397 (2)	0.5747 (3)	0.23806 (7)	0.0211 (4)	
H12A	0.8546	0.5054	0.2700	0.032*	
H12B	0.7591	0.6680	0.2403	0.032*	
H12C	0.9366	0.6355	0.2319	0.032*	
C13	0.1734 (2)	0.6361 (3)	0.15954 (8)	0.0217 (4)	
C14	0.1070 (3)	0.6105 (3)	0.10732 (8)	0.0288 (5)	
H14A	0.0412	0.7163	0.0968	0.043*	
H14B	0.0451	0.4973	0.1048	0.043*	
H14C	0.1901	0.6006	0.0851	0.043*	

### Atomic displacement parameters (Å^2^)

**Table:**

	*U*^11^	*U*^22^	*U*^33^	*U*^12^	*U*^13^	*U*^23^
Ru1	0.01023 (7)	0.00908 (8)	0.01235 (7)	−0.00028 (5)	0.00080 (5)	−0.00063 (5)
Cl1	0.01604 (19)	0.0128 (2)	0.01920 (19)	−0.00040 (15)	0.00236 (15)	0.00325 (16)
Cl2	0.01206 (18)	0.0203 (2)	0.01555 (18)	0.00113 (16)	−0.00022 (14)	0.00109 (16)
N1	0.0121 (6)	0.0121 (7)	0.0151 (6)	−0.0007 (6)	0.0004 (5)	0.0006 (6)
N2	0.0123 (6)	0.0142 (8)	0.0171 (7)	−0.0003 (6)	0.0020 (5)	0.0001 (6)
N3	0.0387 (11)	0.0370 (12)	0.0282 (9)	0.0033 (9)	−0.0009 (8)	0.0035 (8)
C1	0.0102 (7)	0.0168 (9)	0.0185 (8)	−0.0008 (7)	0.0004 (6)	0.0010 (7)
C2	0.0173 (8)	0.0149 (9)	0.0219 (8)	−0.0046 (7)	0.0021 (7)	−0.0027 (7)
C3	0.0211 (9)	0.0113 (9)	0.0223 (8)	−0.0023 (7)	0.0058 (7)	−0.0005 (7)
C4	0.0176 (8)	0.0112 (9)	0.0189 (8)	−0.0008 (7)	0.0049 (6)	0.0039 (7)
C5	0.0163 (8)	0.0147 (9)	0.0170 (8)	−0.0016 (7)	0.0034 (6)	0.0032 (7)
C6	0.0181 (8)	0.0191 (10)	0.0186 (8)	−0.0017 (7)	0.0056 (7)	−0.0023 (7)
C7	0.0141 (8)	0.0170 (9)	0.0237 (9)	−0.0012 (7)	0.0044 (7)	−0.0043 (7)
C8	0.0101 (7)	0.0124 (9)	0.0233 (8)	−0.0008 (6)	0.0011 (6)	−0.0007 (7)
C9	0.0134 (7)	0.0136 (9)	0.0163 (8)	−0.0010 (7)	0.0002 (6)	0.0028 (7)
C10	0.0226 (9)	0.0182 (10)	0.0168 (8)	0.0030 (8)	0.0059 (7)	−0.0013 (7)
C11	0.0140 (7)	0.0157 (9)	0.0156 (8)	0.0005 (7)	0.0029 (6)	0.0032 (7)
C12	0.0258 (9)	0.0201 (10)	0.0172 (8)	−0.0038 (8)	0.0018 (7)	−0.0045 (7)
C13	0.0192 (9)	0.0161 (10)	0.0304 (10)	0.0005 (7)	0.0059 (7)	−0.0002 (8)
C14	0.0274 (10)	0.0304 (12)	0.0281 (10)	−0.0060 (9)	0.0001 (8)	−0.0094 (9)

### Geometric parameters (Å, °)

**Table:**

Ru1—N1	2.0303 (15)	C5—C6	1.523 (2)
Ru1—N2	2.0418 (15)	C5—H5	0.9500
Ru1—C1	2.2082 (16)	C6—C7	1.545 (3)
Ru1—C8	2.2116 (17)	C6—H6A	0.9900
Ru1—C4	2.2154 (17)	C6—H6B	0.9900
Ru1—C5	2.2225 (17)	C7—C8	1.517 (2)
Ru1—Cl1	2.4212 (4)	C7—H7A	0.9900
Ru1—Cl2	2.4265 (4)	C7—H7B	0.9900
N1—C9	1.134 (2)	C8—H8	0.9500
N2—C11	1.136 (2)	C9—C10	1.455 (2)
N3—C13	1.133 (3)	C10—H10A	0.9800
C1—C8	1.385 (3)	C10—H10B	0.9800
C1—C2	1.520 (3)	C10—H10C	0.9800
C1—H1	0.9500	C11—C12	1.459 (3)
C2—C3	1.541 (3)	C12—H12A	0.9800
C2—H2A	0.9900	C12—H12B	0.9800
C2—H2B	0.9900	C12—H12C	0.9800
C3—C4	1.517 (2)	C13—C14	1.452 (3)
C3—H3A	0.9900	C14—H14A	0.9800
C3—H3B	0.9900	C14—H14B	0.9800
C4—C5	1.379 (3)	C14—H14C	0.9800
C4—H4	0.9500		
			
N1—Ru1—N2	163.15 (6)	C3—C4—Ru1	110.84 (12)
N1—Ru1—C1	78.95 (6)	C5—C4—H4	118.0
N2—Ru1—C1	115.42 (6)	C3—C4—H4	118.0
N1—Ru1—C8	114.75 (6)	Ru1—C4—H4	87.0
N2—Ru1—C8	78.91 (6)	C4—C5—C6	123.22 (16)
C1—Ru1—C8	36.53 (7)	C4—C5—Ru1	71.62 (10)
N1—Ru1—C4	77.55 (6)	C6—C5—Ru1	112.56 (12)
N2—Ru1—C4	112.38 (6)	C4—C5—H5	118.4
C1—Ru1—C4	80.14 (7)	C6—C5—H5	118.4
C8—Ru1—C4	94.86 (7)	Ru1—C5—H5	85.9
N1—Ru1—C5	113.76 (7)	C5—C6—C7	114.42 (15)
N2—Ru1—C5	77.06 (6)	C5—C6—H6A	108.7
C1—Ru1—C5	87.90 (7)	C7—C6—H6A	108.7
C8—Ru1—C5	80.42 (7)	C5—C6—H6B	108.7
C4—Ru1—C5	36.21 (7)	C7—C6—H6B	108.7
N1—Ru1—Cl1	83.74 (4)	H6A—C6—H6B	107.6
N2—Ru1—Cl1	87.20 (4)	C8—C7—C6	114.01 (15)
C1—Ru1—Cl1	90.62 (5)	C8—C7—H7A	108.8
C8—Ru1—Cl1	87.59 (5)	C6—C7—H7A	108.8
C4—Ru1—Cl1	160.38 (5)	C8—C7—H7B	108.8
C5—Ru1—Cl1	161.74 (5)	C6—C7—H7B	108.8
N1—Ru1—Cl2	83.88 (4)	H7A—C7—H7B	107.6
N2—Ru1—Cl2	82.78 (4)	C1—C8—C7	124.02 (17)
C1—Ru1—Cl2	161.26 (5)	C1—C8—Ru1	71.60 (10)
C8—Ru1—Cl2	161.37 (5)	C7—C8—Ru1	110.66 (11)
C4—Ru1—Cl2	88.94 (5)	C1—C8—H8	118.0
C5—Ru1—Cl2	92.25 (5)	C7—C8—H8	118.0
Cl1—Ru1—Cl2	94.926 (15)	Ru1—C8—H8	87.7
C9—N1—Ru1	171.31 (14)	N1—C9—C10	179.11 (19)
C11—N2—Ru1	170.70 (15)	C9—C10—H10A	109.5
C8—C1—C2	122.84 (16)	C9—C10—H10B	109.5
C8—C1—Ru1	71.87 (10)	H10A—C10—H10B	109.5
C2—C1—Ru1	113.29 (12)	C9—C10—H10C	109.5
C8—C1—H1	118.6	H10A—C10—H10C	109.5
C2—C1—H1	118.6	H10B—C10—H10C	109.5
Ru1—C1—H1	85.0	N2—C11—C12	178.3 (2)
C1—C2—C3	114.21 (15)	C11—C12—H12A	109.5
C1—C2—H2A	108.7	C11—C12—H12B	109.5
C3—C2—H2A	108.7	H12A—C12—H12B	109.5
C1—C2—H2B	108.7	C11—C12—H12C	109.5
C3—C2—H2B	108.7	H12A—C12—H12C	109.5
H2A—C2—H2B	107.6	H12B—C12—H12C	109.5
C4—C3—C2	113.72 (15)	N3—C13—C14	179.2 (2)
C4—C3—H3A	108.8	C13—C14—H14A	109.5
C2—C3—H3A	108.8	C13—C14—H14B	109.5
C4—C3—H3B	108.8	H14A—C14—H14B	109.5
C2—C3—H3B	108.8	C13—C14—H14C	109.5
H3A—C3—H3B	107.7	H14A—C14—H14C	109.5
C5—C4—C3	123.94 (16)	H14B—C14—H14C	109.5
C5—C4—Ru1	72.17 (10)		
			
N1—Ru1—C1—C8	−168.61 (12)	N1—Ru1—C5—C4	−0.84 (12)
N2—Ru1—C1—C8	2.09 (13)	N2—Ru1—C5—C4	−167.20 (11)
C4—Ru1—C1—C8	112.29 (12)	C1—Ru1—C5—C4	76.11 (11)
C5—Ru1—C1—C8	76.69 (11)	C8—Ru1—C5—C4	112.09 (11)
Cl1—Ru1—C1—C8	−85.11 (10)	Cl1—Ru1—C5—C4	161.71 (12)
Cl2—Ru1—C1—C8	167.48 (12)	Cl2—Ru1—C5—C4	−85.14 (10)
N1—Ru1—C1—C2	72.69 (13)	N1—Ru1—C5—C6	−119.99 (13)
N2—Ru1—C1—C2	−116.61 (13)	N2—Ru1—C5—C6	73.65 (13)
C8—Ru1—C1—C2	−118.70 (18)	C1—Ru1—C5—C6	−43.04 (13)
C4—Ru1—C1—C2	−6.41 (13)	C8—Ru1—C5—C6	−7.06 (13)
C5—Ru1—C1—C2	−42.01 (13)	C4—Ru1—C5—C6	−119.15 (18)
Cl1—Ru1—C1—C2	156.19 (12)	Cl1—Ru1—C5—C6	42.6 (2)
Cl2—Ru1—C1—C2	48.8 (2)	Cl2—Ru1—C5—C6	155.72 (12)
C8—C1—C2—C3	−91.4 (2)	C4—C5—C6—C7	−90.1 (2)
Ru1—C1—C2—C3	−8.64 (19)	Ru1—C5—C6—C7	−7.95 (19)
C1—C2—C3—C4	26.6 (2)	C5—C6—C7—C8	26.4 (2)
C2—C3—C4—C5	50.8 (2)	C2—C1—C8—C7	3.5 (3)
C2—C3—C4—Ru1	−31.22 (18)	Ru1—C1—C8—C7	−102.95 (16)
N1—Ru1—C4—C5	179.21 (11)	C2—C1—C8—Ru1	106.48 (16)
N2—Ru1—C4—C5	13.50 (12)	C6—C7—C8—C1	49.7 (2)
C1—Ru1—C4—C5	−100.05 (11)	C6—C7—C8—Ru1	−31.57 (18)
C8—Ru1—C4—C5	−66.49 (11)	N1—Ru1—C8—C1	12.33 (13)
Cl1—Ru1—C4—C5	−162.97 (11)	N2—Ru1—C8—C1	−178.08 (12)
Cl2—Ru1—C4—C5	95.25 (10)	C4—Ru1—C8—C1	−66.19 (11)
N1—Ru1—C4—C3	−60.44 (12)	C5—Ru1—C8—C1	−99.52 (12)
N2—Ru1—C4—C3	133.85 (12)	Cl1—Ru1—C8—C1	94.30 (10)
C1—Ru1—C4—C3	20.30 (12)	Cl2—Ru1—C8—C1	−167.41 (12)
C8—Ru1—C4—C3	53.86 (13)	N1—Ru1—C8—C7	132.64 (12)
C5—Ru1—C4—C3	120.34 (17)	N2—Ru1—C8—C7	−57.76 (13)
Cl1—Ru1—C4—C3	−42.6 (2)	C1—Ru1—C8—C7	120.32 (18)
Cl2—Ru1—C4—C3	−144.41 (12)	C4—Ru1—C8—C7	54.12 (13)
C3—C4—C5—C6	1.9 (3)	C5—Ru1—C8—C7	20.80 (13)
Ru1—C4—C5—C6	105.41 (17)	Cl1—Ru1—C8—C7	−145.38 (12)
C3—C4—C5—Ru1	−103.53 (17)	Cl2—Ru1—C8—C7	−47.1 (2)

## References

[bb1] Ashworth, T. V., Liles, D. C., Robinson, D. J., Singleton, E., Coville, N. J., Darling, E. & Markwell, J. (1987). *S. Afr. J. Chem.* 40, 183–188.

[bb2] Bruker (2010). *APEX2*, *AXScale* and *SAINT* Bruker AXS Inc., Madison, Wisconsin, USA.

[bb3] Dolomanov, O. V., Bourhis, L. J., Gildea, R. J., Howard, J. A. K. & Puschmann, H. (2009). *J. Appl. Cryst.* **42**, 339–341.

[bb4] Sheldrick, G. M. (2008). *Acta Cryst.* A**64**, 112–122. 10.1107/S0108767307043930 18156677

[bb5] Westrip, S. P. (2010). *J. Appl. Cryst.* **43**, 920–925.

